# Spatiotemporal distribution characteristics and influencing factors of the rate of cardiovascular hospitalization in Ganzhou city of China

**DOI:** 10.3389/fcvm.2023.1225878

**Published:** 2023-12-22

**Authors:** Shanshan Yan, Guoqiu Liu, Xiaoyuan Chen

**Affiliations:** ^1^ School of Public Health and Health Management, Gannan Medical University, Ganzhou, China; ^2^ Key Laboratory of Prevention and Treatment of Cardiovascular and Cerebrovascular Diseases, Ministry of Education, Gannan Medical University, Ganzhou, China

**Keywords:** cardiovascular disease, spatiotemporal distribution, influencing factors, epidemiological characteristics, Ganzhou City

## Abstract

**Aims:**

The objective of this study was to analyze hospitalization rates for cardiovascular diseases (CVD) in Ganzhou City, Jiangxi Province of China from 2015 to 2020 and to uncover the spatiotemporal distribution characteristics and influencing factors, and thus to provide reference for the prevention and control of CVD and public health resources planning.

**Methods:**

The hospitalization data for CVDs from 2016 to 2020 was obtained from the First Affiliated Hospital of Gannan Medical University, and ArcGIS 10.8, SaTScan 9.5, and Matlab 20.0 were used to analyze the spatial autocorrelation, spatiotemporal scan statistics, and potential affecting factors of the hospitalization rates.

**Results:**

The hospitalization rate for CVDs in Ganzhou City showed a slightly increasing trend from 2016 to 2020, with higher rates in winter and summer than that in spring and autumn, and the individuals aged 61 and above constitute a higher proportion compared to other age groups. Additionally, there was a positive correlation between hospitalization rates for CVDs and the counties and districts in Ganzhou City, with high-high aggregation areas mainly distributed in Nankang District, the western urban area of Ganzhou City. The spatial scan analysis identified three different types of significant aggregation areas: high-risk, low-risk, and middle-risk areas. The high-risk area was mainly centered around Zhanggong District or Shangyu County in the central and western regions, with a disease hospitalization rate 2–3 times higher than the rest areas. The study also found that environmental meteorological factors such as the annual average concentration of NO_2_, O_3_, average annual temperature, and annual maximum temperature diurnal range had a significant positive effect on hospitalization rates for CVDs in Ganzhou City, with O_3_ concentration and average annual temperature having significant positive indirect spatial spillover effects.

**Conclusion:**

Winter and summer are the seasons with high hospitalization rate of cardiovascular diseases. County residents aged 61 and above are the higher-risk population that needs to pay more attention on for prevention and control of CVD in Ganzhou City, which exhibits significant spatiotemporal clustering. The urban areas of Zhanggong and Nankang in Ganzhou City are the key areas for prevention and control of CVD. The hospitalization rate of CVD in Ganzhou City is influenced by the aforementioned four environmental meteorological factors, with the annual maximum temperature diurnal range showing the most significant positive direct effect.

## 1. Introduction

CVDs have the characteristics of high incidence and high mortality and have become one of the main diseases affecting human health and the leading cause of human death (1, 2). According to statistics, more than 30% of people worldwide suffer from CVDs, and by 2020, up to 23 million patients will die from CVDs globally. In developed countries such as the United States, the United Kingdom, Canada, Australia, France, and Japan, the mortality rate of CVDs has decreased by more than 50% (3–5). However, CVDs still face enormous challenges in developing countries, accounting for more than 60% of the global disease economic burden (4). CVD related deaths are on the first place in the total death causes of urban and rural residents in China, with 46.74% of deaths in rural areas and 44.26% in cities, and every 2 out of 5 deaths are caused by CVDs (6). Therefore, although the prevention and treatment of CVDs have achieved certain results in developed countries, the number of patients with CVDs in developing countries such as China is still increasing, and there are still serious challenges, with widespread exposure to CVD risk factors among the population in developing countries.

Literature review indicates that CVDs exhibit spatial clustering characteristics in terms of its incidence and mortality distribution (7–9). Meanwhile, the spatial distribution characteristics are significantly influenced by geographical environmental health factors, such as meteorological factors (10–12), atmospheric environmental quality (13–15), social factors (16, 17), and other individual factors (18–20). The mortality and morbidity of cardiovascular diseases are clustered in terms of individual factors such as obesity (19), smoking (21), hypertension (22), physical activity (23), and age (24).

However, the spatial distribution characteristics of CVD and environmental health factors is poorly defined. Endawoke Amsalu (2019) explored the spatial-temporal distribution characteristics of CVD in Beijing, China, (25). Hongyan Ren et al. (2019) found that the two most common CVDs, coronary heart disease and stroke, exhibit spatiotemporal clustering characteristics at the county level in China (26). Almendra (2020) analyzed CVD mortality data from 278 Portuguese continental cities, demonstrating that CVD mortality exhibits significant spatial clustering (27).Geographic Information System (GIS) technology can provide a new perspective and technical method for studying the relationship between cardiovascular diseases and environmental factors in a certain time and space.

From the above literature review, it can be seen that several studies on CVD focus on a large spatial scale and economically developed metropolitan areas in a country. The exploration of the spatial distribution characteristics of CVD in small and medium-sized cities with weaker economic strength and scarce high-quality advanced medical resources should be strengthened, but such regions have not received attention. This gap makes it impossible to identify the spatial characteristics of CVD in economically impoverished small and medium-sized cities, which further limits the accurate prevention and control of CVD in these regions.

Meanwhile, so far, previous studies have shown that the incidence of CVD is closely related to over 300 types of risk factors. (1) Among these, environmental factors have a greater impact on CVD, and the relationship between CVD induction and death and environmental climate factors is closely related globally. Compared to other factors, environmental factors are more observable and therefore more intuitive, making it more effective to avoid, prevent, and control risk factors (11). (2) Various studies have shown that these factors tend to occur simultaneously, increasing the risk of CVD, especially when associated with variations in environmental and climate (28). (3) As mentioned above, the inducing factors of cardiovascular diseases are very complicated. Except for the traditional social and economic factors, medical service conditions and the characteristics of the disease itself, about 15%–20% of patients with cardiovascular diseases have not found the above risk factors. The World Health Organization (WHO) released a report that the death of cardiovascular diseases such as stroke and ischemic heart disease is closely related to the geographical environment, and climate change and air pollution have a greater impact on cardiovascular diseases (12). As a typical spatial data, environmental climate factors have spatial heterogeneity and autocorrelation, and their influence on the hospitalization rate of cardiovascular diseases in Ganzhou is different in different regions. However, at present, it is still blank to explore this scientific problem from the perspective of geographical space by using spatial econometric model.

Ganzhou City is located in the southern area of Jiangxi Province, China. Ganzhou City is divided into 3 districts and 13 counties, and as of the end of 2020, the urbanization rate of the population was 32.5%. Therefore, most of the areas in the region are economically disadvantaged rural areas, and high-quality medical resources are particularly limited. According to a survey by the Ganzhou City Disease Control Center, CVDs are the largest disease affecting the health of the population.

Therefore, the purpose of this study is to explore the spatial distribution characteristics of hospitalized patients with CVD in Ganzhou, and to further analyze the geographic and environmental factors that may influence this distribution from the perspective of geographical space by using spatial econometric model. The results of this study will provide scientific theoretical support for the prevention and control of CVD in economically disadvantaged small and medium-sized cities. In addition, improving their scientific understanding of CVD, reducing the risk of CVD and death at the individual level, and improving the health of residents.

## 2. Materials and methods

### 2.1. Data sources

The data used in this study were obtained from four sources. The diagnosis and hospitalization records of CVD patients in Ganzhou City, Jiangxi Province from 2016 to 2020 were collected and organized from the First Affiliated Hospital of Gannan Medical University, with a time span from January 1, 2016 to December 31, 2020, totaling 14,779 cases. The diagnostic criteria of CVD in this study are coded and classified according to the international classification of diseases, ICD-10 (I00-I99), including: rheumatic heart disease (I00-I09), hypertension and its complications (I10-I15), ischemic heart disease (I20-I25), pulmonary heart disease and pulmonary circulation diseases (I26-I28), cerebrovascular diseases (I60-I69) and other painstaking efforts (29). The hospitalization records included basic information about the patients such as hospitalization number, name, gender, age, marital status, department of treatment, date of onset, diagnosis, length of stay, and home address. Population and economic data for each county and district of Ganzhou City were obtained from the National Bureau of Statistics of China. Electronic map vector data for each county and district of Ganzhou City were obtained from the China Geographic Information Resource Catalogue Service System (http://www.webmap.cn). Temperature, pressure, wind speed, humidity and other data for each county and district of Ganzhou City were obtained from the National Meteorological Data Sharing Service Center and the Ganzhou Meteorological Bureau.

### 2.2. Inclusion and exclusion criteria

Inclusion criteria: Patients with CVD who have lived in Ganzhou City for at least 5 years, aged 25–85 years old. Exclusion criteria: Non-residents of Ganzhou City, patients admitted due to accidents, surgery or other non-CVD related reasons, and patients who were diagnosed with CVD during hospitalization for other reasons.

### 2.3. Research methods

#### 2.3.1. Spatial autocorrelation analysis

Spatial autocorrelation analysis can reflect the spatial distribution of diseases and the strength of association between disease occurrence and surrounding disease occurrence. ArcGIS software was used to perform spatial autocorrelation analysis on CVD in Ganzhou City in 2016, 2018, and 2020.

(1) Global spatial autocorrelation analysis: Global spatial autocorrelation analysis is used to analyze whether there is a correlation hospitalization rate of CVDs in the Ganzhou overall area. The cumulative hospitalization rate is calculated using the number of hospitalization cases and total population in each district of Ganzhou City to reflect the intensity of CVD hospitalization rate. Moran's *I* coefficient is the most commonly used statistic in spatial autocorrelation analysis. The value of Moran's *I* is between −1 and 1. The closer the calculation value is to 1, the stronger the positive spatial correlation ofhospitalization rate of CVDs in the Ganzhou is. The closer the calculation value is to −1, the stronger the negative spatial correlation of hospitalization rate of CVDs in the Ganzhou is. If |*Z*| > 1.96 and Moran's *I* > 0, it indicates that the distribution has a positive global spatial correlation. If |*Z*| > 1.96 and Moran's *I* < 0, it indicates that the distribution has a negative global spatial correlation. If |*Z*| < 1.96, it means that there is no clustering in the overall area, and the distribution is random in space (30).
(2) Local spatial autocorrelation analysis: Local spatial autocorrelation analysis detects the specific location of CVD clustering areas in the Ganzhou area by judging whether the attribute of each spatial location is related to the attribute of neighboring spatial locations. This study used the local spatial autocorrelation analysis statistic LISA to analyze the CVD clustering areas, which are divided into four types: high-high (H-H) clusters, low-low (L-L) clusters, high-low (H-L) clusters, and low-high (L-H) clusters. The first two represents spatial positive correlation areas of hospitalization rate of CVDs in the Ganzhou, while the latter two represent spatial negative correlation areas of hospitalization rate of CVDs in the Ganzhou. Local spatial autocorrelation analysis reflects the degree to which spatial units affect the overall spatial autocorrelation of the study area and identifies spatial clustering points of CVD in Guizhou in each year (30).

#### 2.3.2. Spatio-Temporal scanning

Spatio-temporal scanning analysis can make up for the fact that spatial autocorrelation analysis can not analyze the spatial distribution pattern and scope, and can only reveal the lack of spatial aggregation of data. The statistical analysis of spatio-temporal scanning is based on the scanning statistics of spatial dynamic window, and the Possion model is used to fit the hospitalization data of cardiovascular diseases in Ganzhou. The scanning window is set to be cylindrical (the bottom corresponds to the research area and the height corresponds to the time length), and different times and areas are scanned with the dynamically changing scanning window. According to the actual hospitalization number and expected number of cardiovascular diseases in Ganzhou city inside and outside the scanning window, the Log Likelihood Ratio (LLR) of test statistics is constructed to evaluate whether the number of cases in the window is abnormal or not, and the Relative Risk (RR) is calculated, which can accurately evaluate the risk of each cluster. Monte Carlo method is used to simulate and test the statistical significance of LLR statistics. The LLR value is the largest and the difference is statistically significant, which shows that the risk of cardiovascular disease in this area is the greatest, that is, the scanning window is a kind of aggregation area, and other statistically significant LLR value windows are secondary aggregation areas. In this study, the maximum scanning radius is limited to 30% of the total population at risk, the maximum scanning height is 30% of the total research period, and the scanning window is in months (31).

#### 2.3.3. Spatial durbin model

Because the hospitalization rate of CVDs and environmental climate influencing factors are all spatial data with spatial attributes. Considering the complexity, autocorrelation, and variability of spatial data CVD in Ganzhou, the impact of environmental and climatic risk factors on the hospitalization rate of CVDs in the Ganzhou may vary across different regions. Therefore, the Global Moran's *I* statistic is first used to test the spatial autocorrelation of the data. If there is no spatial autocorrelation, a global linear regression model (OLS regression) can be used for estimation. If there is spatial autocorrelation, spatial econometric models such as spatial lag models, spatial error models, and spatial Durbin models can be used to explore the environmental and climatic risk factors affecting the hospitalization rateof CVDs in Ganzhou.

The spatial Durbin model integrates the characteristics of spatial lag model and spatial error model. It has the advantages of considering both endogenous and exogenous interaction effects between variables, considering the spatial lag effect between independent and dependent variables, providing unbiased coefficient estimates, and nesting both time and space effects. Based on the spatial correlation test, this study will use the spatial Durbin model to analyze the environmental and climatic factors affecting the hospitalization rate of CVDs in Ganzhou. Additionally, in order to further explore the time and space-time joint effects of association between the hospitalization rate of CVDs and environmental climate factors. Referred to model specification in reference 32, this study cites Spatial panel Durbin model with time and space-time lagged dependent variable. The equation can be specified and estimated as follows:

$$y_{it}=y_{i(t-1)}+\rho \overset{N}{\underset{\;j=1}{\sum }}w_{ij}y_{\;jt-1}+\rho \overset{N}{\underset{\;j=1}{\sum }}w_{ij}y_{\;jt}+x_{ij}\beta +\overset{N}{\underset{\;j=1}{\sum }}w_{ij}x_{ij}\theta +u_{i}+u_{t}+\varphi_{it}$$

In Equation, i and *j* denote two different spatial units (Counties) in Ganzhou. t denotes a time point (year). *y* denotes the dependent variable (hospitalization rate of CVDs). x denotes the exogenous independent variables (environmental and climatic risk factors). w denotes a non-negative spatial weight matrix which explains spatial relationships between spatial units. *N* donates the number of spatial units. ρ and λ quantify the spatial spillover effects of spatially lagged dependent variable wy and spatially lagged error term. β denotes the coefficient of *x*. θ quantifies the spatial spillover effects from spatially lagged independent variables wx. $u_{i}$ denotes the individual (spatial) effect and $u_{t}$ denotes the time effect respectively, which could be fixed or random effects. φ and ε denote the error term. *$y_{i(t-1)}$* denotes the time lagged dependent variable with 1-year time interval. $\rho \overset{N}{\underset{\;j=1}{\sum }}w_{ij}y_{\;jt-1}$ denote the space-time lagged dependent variable (32).

## 3. Results

### 3.1. General situation of epidemic

#### 3.1.1. Temporal distribution

The data showed that the average annual hospitalization rate for CVDs in 18 counties and districts of Ganzhou City from 2016 to 2020 was 32.34/100,000, with the highest hospitalization rate reported in 2019 (39.26/100,000) and the lowest in 2016 (27.18/100,000). The number of hospitalization cases for CVDs continued to increase from 2016 to 2020, reaching a relative peak in 2019. Due to the outbreak of the COVID-19 pandemic in 2020 and various policy restrictions on hospitalization, the growth of hospitalization cases for CVD stopped, but the overall development situation still cannot be ignored.

In terms of seasonal distribution, The hospitalization rate for CVD in Ganzhou City during 2016–2020 showed an overall upward trend with fluctuations. The month of June had the highest number of reported hospitalization cases. Hospitalization for CVD mainly occurred in summer (June–August) and winter (December–February), with higher hospitalization rates in winter and summer than in spring and autumn, the results are shown in Table 1.

**Table 1:** Monthly report on hospitalization rate of CVD in Ganzhou from 2016 to 2020.

Month	Year/Case						Total
	2016	2017	2018	2019	2020	Case	Proportion (%)
1	200	217	295	364	178	1,254	8.76%
2	191	214	284	320	191	1,200	8.38%
3	153	200	211	227	217	1,008	7.04%
4	127	234	246	278	240	1,125	7.85%
5	163	227	252	234	258	1,134	7.91%
6	244	264	260	383	234	1,385	9.67%
7	173	229	299	396	284	1,381	9.64%
8	218	242	259	255	240	1,214	8.48%
9	200	158	243	199	197	997	6.96%
10	219	203	228	208	199	1,057	7.38%
11	209	210	332	312	208	1,271	8.87%
12	276	259	230	327	205	1,297	9.05%
Total	2,373	2,657	3,139	3,503	2,651	14,323	100%

#### 3.1.2. Population distribution

In the reported cases of CVD hospitalizations from 2016 to 2020, the male-to-female ratio was 1.27:1. Hospitalizations for CVD in patients aged below 45, 45–60, and above 61 years accounted for 4.18% (619/14,789), 40.8% (6,034/14,789), and 55.01% (8,136/14,789), respectively. The majority of patients were farmers.

### 3.2. Spatial distribution analysis

**Figure 1:**
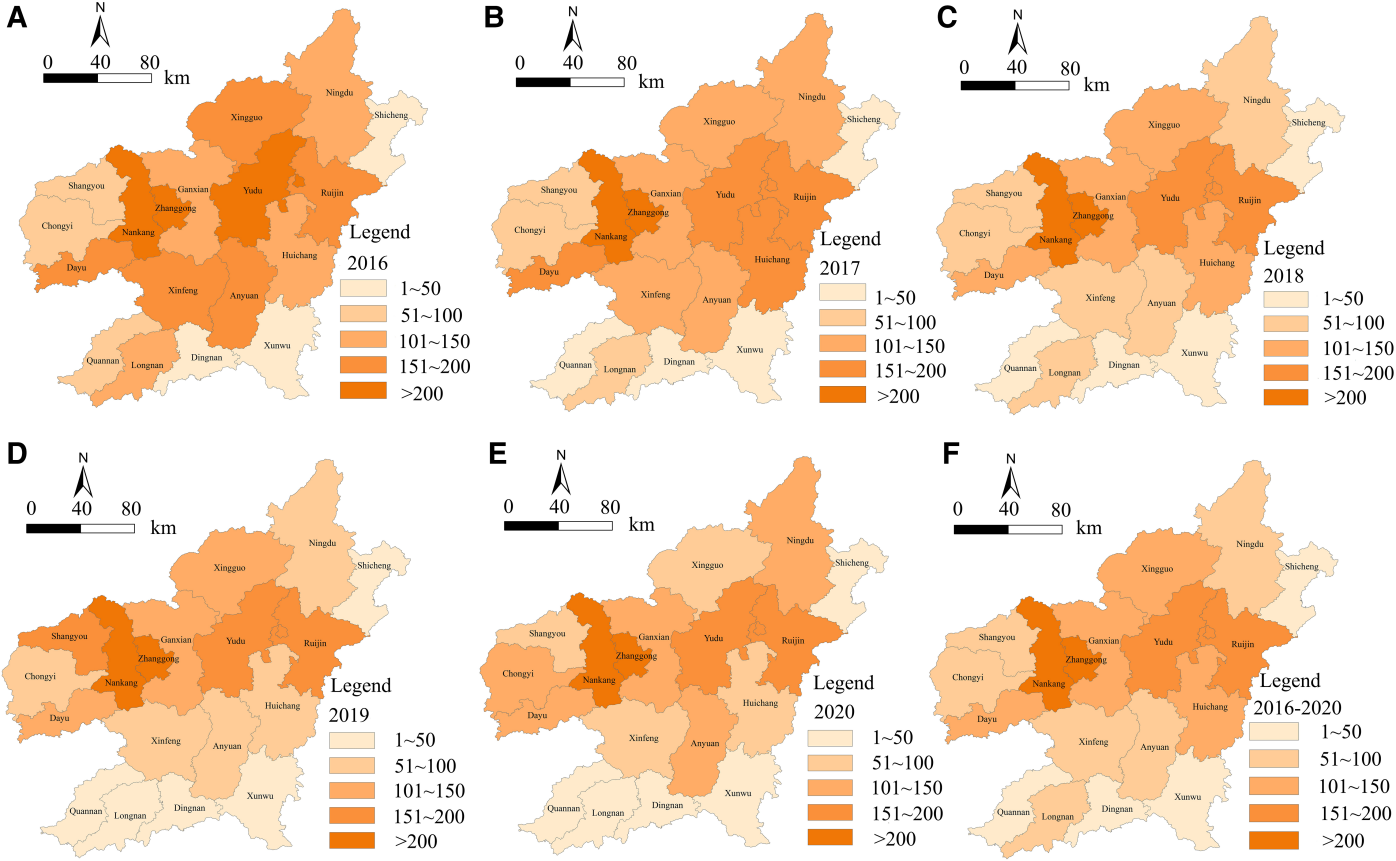
Grade map of cumulative hospitalization rate of CVD cases in Ganzhou from 2016 to 2020. Figures (**A–F**) are based on the data of cardiovascular inpatients in a big data center of a 3A hospital in Ganzhou. The natural segment method of arcgis, a spatial statistical analysis software, is used to visually express the inpatient rate of cardiovascular diseases in Ganzhou from 2016 to 2020, which is divided into five grades, of which the first grade is 1–50 people. The second level is 50–100 people; The third level is 100–150 people; The fourth level is 150–200 people; The fifth grade is >200 people, in which the darker the color in the picture means more hospitalized patients, and the lighter the color means fewer hospitalized patients.

Using ArcGIS software, a cumulative hospitalization rate level map of CVD in Ganzhou City was visualized, where the color depth shows the level of hospitalization rate, with darker colors indicating higher rates. From the map, it can be seen that from 2016 to 2020, all 18 districts and counties in Ganzhou City with the majority of cases concentrated in the two urban districts of Zhanggong and Nankang. The annual spatial distribution showed a gradual expansion from these two urban districts to the west and east of Ganzhou City. The counties of Shicheng, Xunwu, Dingnan, Longnan, and Quannan had relatively low overall hospitalization rates. See Figure 1 for details.

### 3.3. Spatial autocorrelation analysis

#### 3.3.1. Global spatial autocorrelation analysis

The global Moran's *I* coefficient of the hospitalization rate reported in 18 counties (districts, cities) in Ganzhou City was positive, indicating a positive correlation in the spatial distribution of the hospitalization rate of CVD in Ganzhou City. The *P* value was <0.05, indicating that the differences were statistically significant. The hospitalization rate of CVD in Ganzhou City showed significant spatial clustering from 2016 to 2020. Among them, the clustering in 2019 was the highest (Moran's *I* = 0.638817), and the clustering in 2016 was the lowest (Moran's *I* = 0.15). The data analysis results are shown in Table 2.

**Table 2:** Spatial global autocorrelation of CVD in Ganzhou from 2016 to 2020.

Year	Moran-*I*	*Z-*score	*P-*value	Pattern
2016	0.210839	2.329718	0.043611	Clustered
2017	0.321855	2.185182	0.049405	Clustered
2018	0.518399	3.046626	0.002314	Clustered
2019	0.638817	3.695395	0.000220	Clustered
2020	0.439960	2.864839	0.004172	Clustered

#### 3.3.2. Local spatial autocorrelation analysis

**Figure 2:**
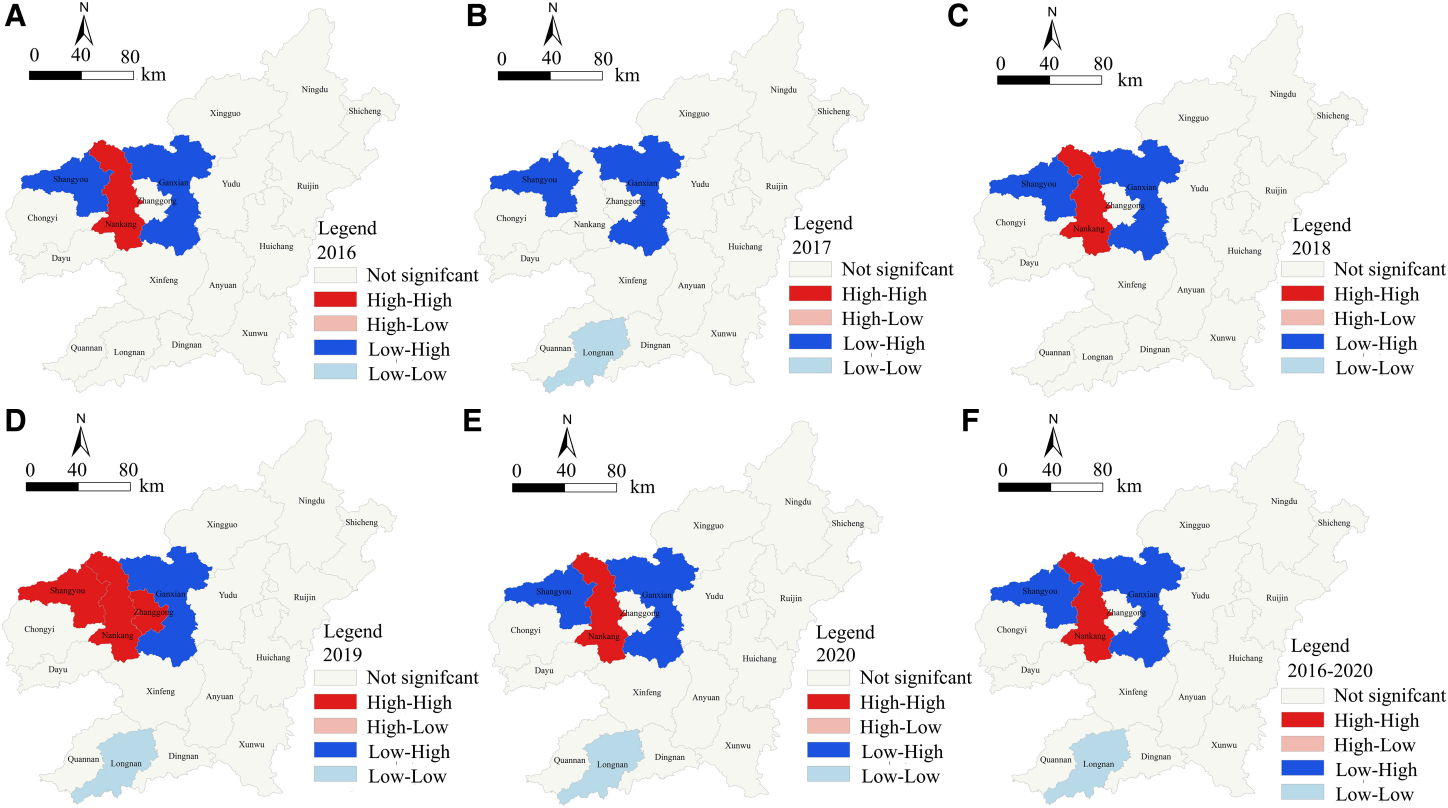
Local spatial autocorrelation map of cumulative hospitalization rate of CVDs in Ganzhou from 2016 to 2020. The data in sub-charts (**A–F**) come from the hospitalization data of cardiovascular patients in a big data center of a 3A hospital in Ganzhou. By using the spatial autocorrelation analysis method of spatial statistical analysis software ArcGIS, the hospitalization rate of cardiovascular diseases in Ganzhou from 2016 to 2020 is analyzed in spatial local autocorrelation. The results are divided into no significant spatial autocorrelation, high-high spatial significant clustering areas, high-low spatial significant clustering areas, low-high spatial significant clustering areas and low-low spatial significant clustering areas. The figures are different.

The results showed that there were three types of statistically significant clusters during the period of 2016–2020: (1) “high-high” clusters mainly distributed in Nankang district; (2) “low-low” clusters mainly distributed in Longnan County; (3) “low-high” clusters mainly distributed in Shangyou County andGuanxian District. From the perspective of temporal evolution, the spatial distribution patterns of the three types of clustering areas did not change much. The “high-high” clustering area only increased from Nankang district to include Zhanggong district and Shangyou County in 2019. At the same time, Longnan County was added as a “low-low” clustering area in 2017, 2019, and 2020. Shangyou County changed from a “low-high” clustering area to a “high-high” clustering area in 2019 (Figure 2).

### 3.4. Spatial-temporal scan analysis

**Figure 3:**
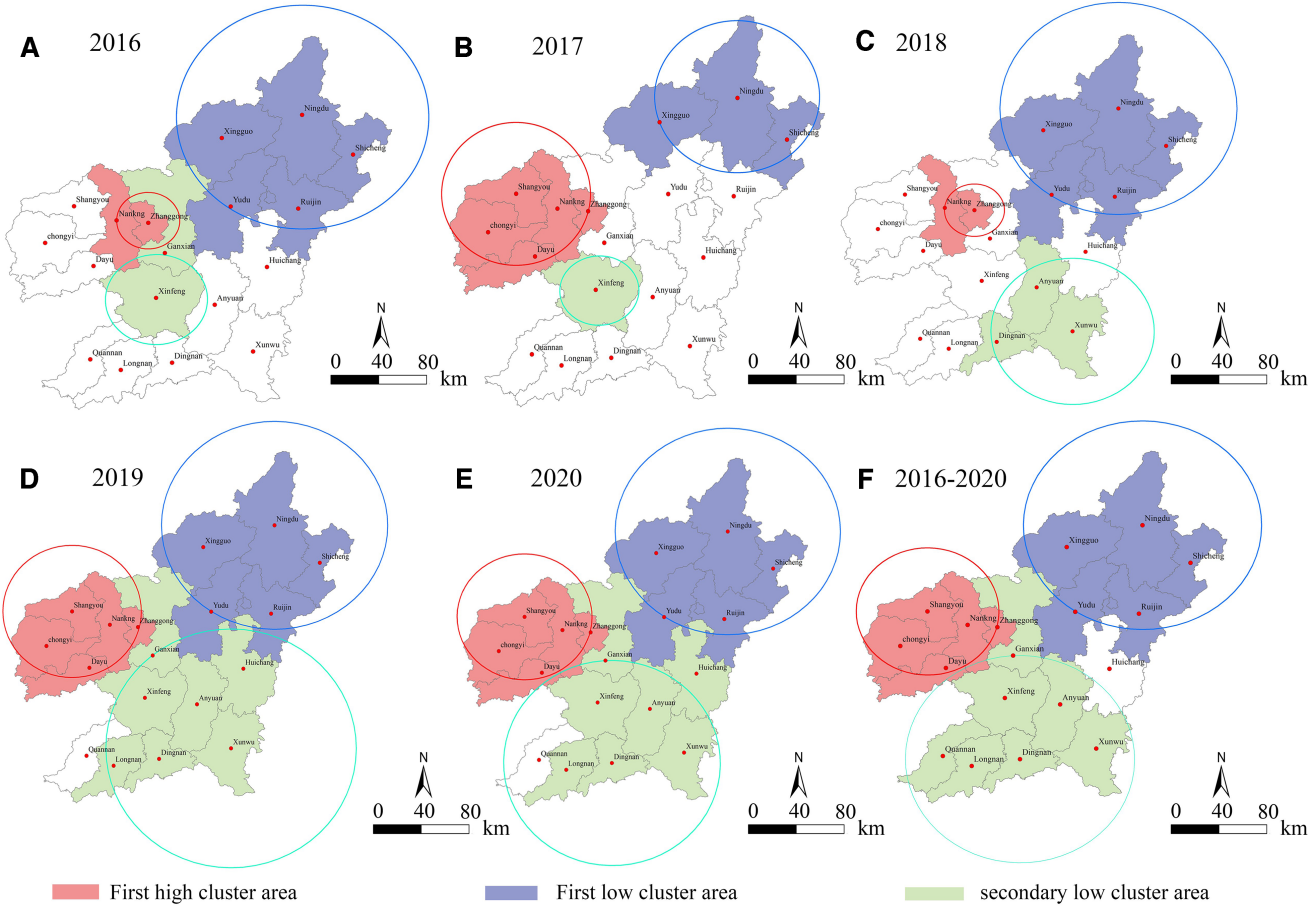
Spatial-temporal scanning analysis chart of inpatients with CVD in Ganzhou from 2016 to 2020. Figures (**A–F**) are based on the data of cardiovascular inpatients in a big data center of a 3A hospital in Ganzhou. Taking *P* < 0.01 as the criterion of significance level, the medical records of cardiovascular inpatients in Ganzhou from 2016 to 2020 were scanned and analyzed by using Sat Scan software. Results Three different types of significant agglomeration areas were scanned in space, namely, one kind of high risk, one kind of low risk and two kinds of low risk. Red, purple and green in the figure were marked as different area types, and the circles with corresponding colors indicated the spatial coverage of different area types.

The results showed from 2016 to 2020, there were three significant cluster areas of CVD hospitalization in Ganzhou City: one high-risk cluster, one low-risk cluster, and one second-tier low-risk cluster. The high-risk cluster was mainly centered on the middle and western regions, including Zhanggong District, Ganxian District, Nankang District, Shangyou County, Chongyi County, and Dayu County. The hospitalization rateof CVD in the high-risk cluster was 2–3 times higher than that in the surrounding areas. The first low-risk cluster was mainly distributed in the northern region, with Nindu County as the center, including five counties and cities such as Shicheng County and Xingguo County. The second-tier low-risk cluster was mainly distributed in the central and southern regions, with Xinfeng County, Xunwu County, or Dingnan County as the center, including seven counties and districts such as Dingnan County and Longnan County. The cluster time spanned from June to August and December to February. See Table 3 and Figure 3 for details.

**Table 3:** Spatial global autocorrelation of CVD in Ganzhou from 2016 to 2020.

Year	Cluster type	Cluster Center	Radiation radius (km)	Number of districts	Begin date	End date	*RR*	*LLR*	*P*
2016	First high cluster	Zhanggong district	22.43	2	2,016.07	2,016.09	2.80	165.57	<0.01
	First low cluster	Ningdu county	88.21	5	2,016.06	2,016.12	0.50	70.16	<0.01
	Secondary low cluster	Xinfeng county	36.09	2	2,016.07	2,016.11	0.60	16.10	<0.01
2017	First high cluster	Shangyou county	53.99	5	2,017.06	2,017.01	1.94	75.60	<0.01
	First low cluster	Ningdu county	59.25	3	2,017.07	2,017.02	0.45	41.05	<0.01
	Secondary low cluster	Xinfeng county	0.00	1	2,017.06	2,017.12	0.39	17.32	<0.01
2018	First high cluster	Zhanggong district	22.43	2	2,018.06	2,018.11	2.82	211.53	<0.01
	First low cluster	Ningdu county	88.21	5	2,018.07	2,018.12	0.51	79.87	<0.01
	Secondary low cluster	Xunwu county	52.32	3	2,018.07	2,018.12	0.52	65.61	<0.01
2019	First high cluster	Shangyou county	53.99	5	2,019.06	2,019.11	2.82	496.71	<0.01
	First low cluster	Ningdu county	88.21	5	2,019.01	2,019.05	0.33	259.25	<0.01
	Secondary low cluster	Xunwu county	99.97	7	2,019.01	2,019.06	0.47	144.98	<0.01
2020	First high cluster	Shangyou county	53.99	5	2,020.12	2,020.02	3.75	378.07	<0.01
	First low cluster	Ningdu county	88.21	5	2,020.08	2,020.12	0.39	98.16	<0.01
	Secondary low cluster	Xunwu county	99.97	7	2,020.08	2,020.11	0.44	52.37	<0.01
2016–2020	First high cluster	Shangyou county	53.99	5	2,018.11	2,020.02	3.29	2,029.61	<0.01
	First low cluster	Ningdu county	88.21	5	2,016.04	2,018.09	0.48	462.23	<0.01
	Secondary low cluster	Dingnan county	86.94	7	2,016.08	2,019.01	0.58	220.60	<0.01

In different years (Figure 3), a significant high-risk cluster was identified with a radius of 22.43 km centered on Zhanggong District, covering Zhanggong District and Ganxian District, where the risk of hospitalization due to CVD was 2.80 times higher than in other areas (RR = 2.80, LLR = 165.57, *P* < 0.00) in 2016. In addition, a level 1 low-risk cluster centered on Ningdu County with a radius of 88.21 km and covering five counties and districts, and a level 2 low-risk cluster centered on Xinfeng County with a radius of 36.09 km and covering Xinfeng County and Ganxian District. The spatiotemporal scan analysis results were consistent with the results of the local spatial autocorrelation analysis. The spatial distribution of the significant high-risk cluster and level 1 low-risk cluster in 2016 was completely consistent with that in 2018. However, the significant level 2 low-risk cluster in 2018 showed a different spatial distribution, centered on Xunwu County in the southeast, radiating to Anyuan County and Dingnan County.

In addition, the spatial distribution characteristics of the high-risk areas of type 1 were completely consistent in 2017, 2019, 2020, and 2016–2020, showing a high-risk aggregation center in the western Shangyou County, with a radius of 53.99 km, The hospitalization rate of CVDs in these aggregation areas was mostly 2–3 times higher than that in surrounding areas in most of the years analyzed (RR ranging from 1.94 to 3.29). As for the distribution characteristics of the significantly low-risk areas, the areas of low risk in 2019, 2020, and 2016–2020 continued to increase, and the two different levels of low-risk aggregation areas had similar spatial distribution patterns. Type 1 low-risk aggregation area was centered on the northern Ningdu County, with a radius of 88.21 km. Type 2 low-risk aggregation area was centered on the southern Xunwu County or Dingnan County, with a radius of about 90 km. In addition, the areas of type 1 and type 2 in 2017 were relatively small.

### 3.5. Analysis of influencing factors

So far, previous studies have shown that the incidence of CVD is closely related to over 300 types of risk factors (33). Among these, environmental factors have a greater impact on CVD, and the relationship between CVD induction and death and environmental climate factors is closely related globally (34). Therefore, this study mainly focuses on analyzing the impact of environmental health factors on CVD from a health geography perspective.

Based on previous research results, this study mainly explores environmental risk factors from two aspects: meteorology and atmospheric pollution.

Initially selected five indicators of air pollution risk factors, including annual average concentrations of PM_2.5_ (35), PM_10_ (36), SO_2_ (37), NO_2_ (38), and O_3_ (39), and seven indicators of climate risk factors, including annual average temperature (40), annual maximum temperature difference (12), annual average air pressure (28), annual average wind speed (28), annual average relative humidity, annual average precipitation, and annual average sunshine hours (11), as explanatory variables for the dependent variable, the hospitalization rate of CVDs. This study used stepwise regression to screen the 12 explanatory variables and finally selected annual average concentrations of NO_2_ and O_3_, annual average temperature, and annual maximum temperature difference as environmental health impact factors for CVDs.

Before model estimation, the global Moran's *I* index of each variable was calculated to test for spatial autocorrelation. The results showed that the Moran's *I* index of the dependent variable, CVD hospitalization rate, had a *Z* statistic greater than the critical value of 2.576 and a significance level less than 1%, indicating significant spatial autocorrelation. With the exception of yearly maximum temperature diurnal range, all explanatory variables passed the significance test, indicating that the dependent variable had spatial clustering characteristics. Therefore, spatial variables need to be introduced into the non-spatial regression model, i.e., spatial econometric models need to be used.

This study used the spatial Durbin model and the Matlab software to estimate the spatial regression model of CVD hospitalization rate, and the estimation results are shown in Table 4. The adjusted *R*-squared value is 0.984, indicating that the overall fitting effect of the regression model is good, and the overall regression reliability is relatively high. The results show:

(1) The spatial lag coefficient of the dependent variable *y*, the hospitalization rate of CVDs, is 0.725 and significant at the 1% level, indicating the presence of spatial spillover effects in the spatial model of CVD hospitalization rate. Changes in CVD hospitalization rates in one area will bring about changes in adjacent areas' rates, given that other influencing factors remain constant. On average, if the hospitalization rate of CVDs in the adjacent areas increases by 1%, the hospitalization rate in the local area will increase by 0.725%. In addition, the estimated coefficients of the explanatory variable spatial lag terms W × lx2 and W × lx3 are significant at the 1% and 5% levels, respectively.
(2) Based on the analysis of direct effects from Table 5, the regression coefficients of the four explanatory variables are all positive and pass the significance test, consistent with previous theoretical analysis. The direct effect of the explanatory variable nitrogen dioxide (NO_2_) is 0.087 and significant at the 1% level, indicating a significant positive effect of nitrogen dioxide concentration on CVD hospitalization rate. Holding other risk factors constant, a 1% increase in nitrogen dioxide concentration will lead to a 0.087 percentage point increase in CVD hospitalization rate. The direct effect of the explanatory variable ozone concentration (O_3_) is 0.098 and significant at the 5% level, indicating a positive effect of ozone concentration on CVD hospitalization rate. Holding other risk factors constant, a 1% increase in ozone concentration will lead to a 0.098 percentage point increase in CVD hospitalization rate. The direct effect of average temperature is 0.572 and significant at the 1% level, indicating a positive effect of average temperature on CVD hospitalization rate. Holding other risk factors constant, a 1% increase in average temperature will lead to a 0.572 percentage point increase in CVD hospitalization rate. The explanatory variable with the largest direct effect is the maximum temperature difference, with a direct effect value of 3.683 and significant at the 1% level, indicating that the maximum temperature difference is the most important environmental climate risk factor for CVD hospitalization rate, with a direct effect significantly higher than other environmental climate factors. A 1% increase in the maximum temperature difference will lead to a 3.683 percentage point increase in CVD hospitalization rate.
(3) From the indirect effects or spatial spillover effects shown in Table 5, only the explanatory variables O_3_ concentration and average temperature have passed the significance test at the 1% and 5% levels, respectively, indicating that O_3_ concentration and average temperature have spatial spillover effects. Among them, O_3_ concentration shows a positive spatial spillover effect, indicating that an increase in O_3_ concentration in one area has a positive effect on the CVD hospitalization rate in adjacent areas, which also shows an increasing trend. Similarly, average temperature also shows a positive spatial spillover effect, indicating that an increase in average temperature in one area also brings an increase in the CVD hospitalization rate in adjacent areas. However, the spatial spillover effect of average temperature is significantly higher than that of O_3_ concentration, indicating that the meteorological factor of average temperature has a significantly greater spatial spillover effect on adjacent areas than the environmental pollution factor of O_3_ concentration.

**Table 4:** Spatial durbin model regression results of influencing factors of hospitalization rate of CVD.

Variable	Coefficient	*t*-Statistic	Variable	Coefficient	*t*-Statistic
X1	−0.057^b^	−2.171	*W *× *X1*	−0.019	−0.512
X2	0.091^a^	0.972	*W *× *X2*	0.022^b^	2.135
X3	0.488^b^	2.266	*W *× *X3*	0.197^a^	6.581
X4	3.162^a^	26.83	*W *× *X4*	−0.414	−1.938
Constant term	−5.376^a^	−6.691			
rho	0.725^a^	3.778			
R^2^	0.984				
Log-L	31.531				

X1 (annual average concentration of NO2); X2 (annual average concentration of O3); X3 (annual average temperature); X4 (daytime temperature difference between the annual maximum and minimum).

^a^Indicates significance at 1% level. ^b^Indicates significance at 5% level.

**Table 5:** Effect decomposition estimation results of spatial durbin model in hospitalization rate of CVD.

Variable	Direct effect	*t-*Statistic	Indirect effect	*t-*Statistic	Total effect	*t-*Statistic
X1	0.087^b^	4.862	0.006	1.249	0.093^a^	2.151
X2	0.098^a^	2.201	0.032^a^	2.221	0.130^a^	2.218
X3	0.572^b^	4.563	0.206^b^	5.219	0.778^b^	6.201
X4	3.683^b^	6.521	−0.453	0.832	3.230^a^	2.217

X1 (annual average concentration of NO2); X2 (annual average concentration of O3); X3 (annual average temperature); X4 (daytime temperature difference between the annual maximum and minimum).

^a^Indicates significance at 1% level.

^b^Indicates significance at 5% level.

## 4. Conclusion and discussion

CVD incidence, hospitalization, and mortality exhibit significant spatial clustering and heterogeneity. Spatial econometric methods provide new techniques and means for identifying spatial features of CVD and studying the spatial effects of influencing factors. Therefore, in this study, hospitalization data for CVD in Ganzhou City, Jiangxi Province from 2015 to 2020 were collected, and descriptive statistical analysis was used to conduct an epidemiological triad analysis of hospitalization rates. For the first time, spatial autocorrelation analysis, spatiotemporal scan statistical analysis, and spatial effect analysis of influencing factors were performed on CVD hospitalization rates at the city-level spatial scale in China.

The study found that the average annual hospitalization rate for CVD in 18 counties and districts in Ganzhou City from 2016 to 2020 was 32.34/100,000, showing a fluctuating upward trend, indicating that the prevention and control situation is still severe. The increase in CVD hospitalization rate is mainly due to the significant increase in various risk factors, such as hypertension, diabetes, obesity, smoking, climate change, air pollution, etc., which has been confirmed in other CVD studies. Therefore, Ganzhou City needs to further emphasize the prevention and control of CVD and increase the publicity of prevention and control knowledge. The hospitalization rate in winter and summer is significantly higher than that in spring and autumn, which is consistent with the conclusions of previous researchers (12, 40). The reasons for this are as follows: first, CVD is significantly affected by seasonal temperature and other meteorological factors. Physiologically, cold temperatures cause blood vessel constriction, blood pressure elevation, and indoor hypoxia, which may lead to congenital arterial ductal closure and reduce the intake of trace elements such as zinc, chromium, manganese, selenium, cadmium, and lead. These physiological effects can accelerate the occurrence of CVD and result in hospitalization. Second, in winter and summer, the humidity is relatively high, and the high humidity environment reduces the body's ability to transport and dissipate heat. Sweating is an important way for our body to maintain normal internal and external circulation and body temperature. A humid environment affects the sweating process and is not conducive to maintaining body temperature, which has a serious impact on the occurrence of CVD. The hospitalization rate in other months fluctuates little at a relatively constant incidence level. Third, for CVD, sudden temperature rise and fall and large temperature differences in winter and summer can cause blood vessel constriction, blood pressure elevation, and ultimately trigger acute CVD. The diurnal temperature range is significantly positively correlated with the risk of CVD death.

In terms of population distribution, the number of male inpatients is higher than that of female inpatients, which is consistent with other research findings (20). This may be due to the fact that men are more likely to be the economic backbone of their families, have higher work intensity, and engage in more risky behaviours such as smoking and drinking, leading to a higher incidence of CVD and hospitalization. The individuals aged 61 and above constitute a higher proportion compared to other age groups. Related literature studies have also shown that more than half of the cases of CVD and 80% of CVD deaths occur in adults over 61 years old, and aging and the extension of life expectancy will have a significant impact on the burden of CVD in the elderly (24). In addition, farmers have become a high-risk group for CVD due to the lack of high-quality medical resources and low awareness of health care. Therefore, it is important to focus on strengthening cardiovascular monitoring and prevention work for elderly farmers over 61 years old, in order to reduce the economic burden of CVD.

The global spatial autocorrelation analysis of the CVD hospitalization rate in Ganzhou City showed that the hospitalization rate exhibited significant spatial clustering from 2016 to 2020, which did not conform to the characteristics of random distribution. This is consistent with the results of other researchers who used spatial autocorrelation analysis to study this disease (7, 8, 26, 27, 41). However, since global spatial autocorrelation analysis cannot provide precise location information, this study further used local spatial autocorrelation analysis. The results showed that the Zhonggong District in the western administrative district of Ganzhou City and Nankang City were high-risk areas for CVD, while Longnan County in the southern part of Ganzhou City was a cold spot for CVD. The spatiotemporal scan analysis revealed three significant clustering areas for CVD: one high-risk, one low-risk, and one secondary low-risk area. The high-risk area was mainly centered in the Zhonggong District or Shangyu County in the central and western regions, with a morbidity rate 2–3 times higher than that of surrounding areas. The low-risk area was mainly distributed in the northern region, centered around Ningdu County, and the majority of the clustering occurred during the autumn and winter seasons from October to December each year.

In conclusion, the high-risk clustering areas were mainly concentrated in Zhanggong District and Nankang City, which are densely populated areas with high social and economic development levels and significant environmental pollution, consistent with other reported results. For example, Endawoke A, Mengyang L, et al. found that the high-risk clustering areas for cardiovascular hospitalizations in Beijing included the central area of Xicheng District (25). Poliany CO, Rodrigues ES, et al. found that the high mortality rate of CVDs in the two Brazilian cities of Cuiabá and Governador Valadares was clustered in the core areas with high income ratios (42). The high hospitalization rates in Zhanggong District and Nankang City may be due to several factors, including the fact that the two areas are located in a low mountainous area surrounded by mountains, and are traversed by the Zhang, Gong, and Gan rivers, with significant temperature changes and high humidity, which may lead to more prominent CVDs. Additionally, the two areas have high economic levels and high-income ratios, which may lead to excess nutrition and obesity, which can increase the hospitalization rate for CVDs. Moreover, most low-income families are located in the underprivileged counties under the jurisdiction of Ganzhou City, which have limited access to healthcare opportunities and face significant economic burdens from CVDs. Furthermore, Shangyou County was identified as a high-risk area for CVDs, which may be related to the aging population. According to the statistical yearbook of Jiangxi Province, the proportion of people aged 60 and above in Shangyou County was 18.16% in 2022, which is much higher than the average of 15.04% for the aging population in Ganzhou City, and the proportion of elderly people in Shangrao City is still expanding.

The above three areas should be the key focus of prevention and control measures for CVDs. Based on the characteristics of the affected population and peak incidence period, risk assessment of CVDs should be carried out in these two areas. A second-level prevention and control strategy should be implemented, which is based on the identification of risk factors, early detection, diagnosis and treatment. Furthermore, it was found that the coverage of low-risk aggregation areas is gradually expanding, with a lower reporting rate. This may be due to the fact that the low-risk areas are mainly located in the middle and south of the region, which are mainly mountainous and hilly areas that can act as a barrier, with a warm and humid monsoon climate, and low humidity, which may lead to a relatively low hospitalization rate for CVDs. Primary prevention and control strategies should be implemented in these areas to prevent the potential spread of diseases and the occurrence of the “tip of the iceberg” phenomenon.

As mentioned earlier, a large body of previous research has shown that environmental and climatic factors have a significant impact on CVD. Considering the complexity, autocorrelation, and variability of spatial data, the effect of environmental and climatic factors as explanatory variables on the dependent variable of CVD hospitalization rate has different spatial effects in different regions. The results of this study also showed that there is spatial autocorrelation between the data, and spatial variables need to be introduced into non-spatial regression models to explore the influencing factors of CVD hospitalization rate and their spatial effects. However, existing research on CVD still uses traditional statistical analysis models that do not consider spatial effects to analyse the influencing factors of CVD. Therefore, this study is the first to use a spatial Durbin model to explore the influencing factors and spatial effects of CVD hospitalization rate, which to some extent, complements the methodological system of CVD-related research.

Ganzhou City belongs to the subtropical monsoon climate zone, with an average annual temperature between 19.1 and 20.8 °C in each county, especially the highest daytime temperature in summer is generally above 36 °C, which belongs to a high-temperature area. The daily temperature difference is also large, up to 13 °C. The spatial Durbin model study found that in terms of meteorological risk factors, temperature has a significant positive impact on the hospitalization rate of CVDs in Ganzhou City. Related studies also show that CVDs are generally more common in seasons with low or high temperatures, such as summer and winter (40). Pathological studies of CVDs have shown that when the environment is cold, peripheral blood vessels contract, which raises blood pressure and can cause spasms or blood clots, leading to CVDs. In addition, low temperatures can also weaken the body's immune system, thereby increasing the incidence of CVDs. Conversely, high temperatures can also lead to an increase in the incidence of CVDs (40). High temperatures can increase blood viscosity and cholesterol levels, leading to increased cardiac workload and blood clotting. This study also found that the maximum daily temperature difference has an extremely significant positive impact on the hospitalization rate of CVDs in Ganzhou City (12). The hospitalization rate of CVDs in each county of Ganzhou City increases with the increase of the annual temperature difference. For CVDs, sudden changes in external temperature can affect blood vessel contraction, raise blood pressure, and ultimately lead to acute CVDs. Related studies have found a positive correlation between large temperature differences and acute stroke deaths.

In terms of atmospheric pollution, both nitrogen dioxide (NO_2_) and ozone (O_3_) concentration have a significant positive impact on the hospitalization rate of CVDs. Keeping other risk factors constant, an increase of 1% in carbon dioxide concentration directly leads to an increase of 0.087 percentage points in the hospitalization rate of CVDs. Similarly, an increase of 1% in ozone concentration directly leads to an increase of 0.098 percentage points in the hospitalization rate of CVDs. Meanwhile, ozone concentration (O_3_) also shows positive spatial spillover effects, that is, as the ozone concentration (O_3_) in one area increases, the hospitalization rate of CVDs in adjacent areas also shows an increasing trend. Both of these atmospheric pollutants mainly affect the circulatory system by damaging endothelial cells, accelerating blood coagulation, causing autonomic nervous system disorders, and triggering oxidative stress and inflammatory responses, leading to an increased risk of CVDs (37–39).

Therefore, the local government of Ganzhou should take effective protective measures to address the CVD risk caused by atmospheric pollution. The following recommendations are suggested: first, continue to optimize Ganzhou's energy structure, vigorously develop and utilize new energy such as wind energy, biomass energy, and solar energy, in order to further optimize the energy structure and improve air quality; second, focus on renovating high-pollution, high-energy-consuming, and low-efficiency industries and enterprises, promote regional resource recycling transformation. Furthermore, key prevention and control measures should be taken for dust pollution caused by construction sites, road dust, industrial enterprise dust emissions, and material storage and handling. For example, dust control measures such as sprinkling, spraying, and dust collection should be carried out during different time periods; third, strengthen the control of VOCs and NOx emissions, enhance the control of enterprises and industrial sources, increase the efforts on vehicle exhaust control and testing, and restrict personal ownership of automobiles. In addition, targeted measures should be taken to address the seasonal characteristics of O_3_ pollution, such as implementing staggered production and operation schedules for enterprises, and appropriately increasing the night time working hours for outdoor work in spring and autumn while shortening the daytime working hours.

In addition, this study has some limitations. The data on CVD hospitalization rates in Ganzhou City mainly comes from the case data of the First Affiliated Hospital of Gannan Medical University, which can only reflect the situation of some CVD hospitalizations in Ganzhou City and cannot comprehensively show the overall CVD hospitalization rate in Ganzhou City, leading to underestimation of the CVD hospitalization rate in Ganzhou City. On the other hand, as previously mentioned, the incidence of CVD is influenced by multiple factors. This study only explores the impact and spatial effects of external environmental climate hazards, based on the medical geography perspective, without analyzing the internal medical mechanisms. Future studies need to comprehensively and systematically study both internal and external factors.

## Data Availability

The original contributions presented in the study are included in the article/Supplementary Material, further inquiries can be directed to the corresponding author.
